# Pathophysiology of Circulating Biomarkers and Relationship With Vascular Aging: A Review of the Literature From VascAgeNet Group on Circulating Biomarkers, European Cooperation in Science and Technology Action 18216

**DOI:** 10.3389/fphys.2021.789690

**Published:** 2021-12-14

**Authors:** Kristina R. Gopcevic, Eugenia Gkaliagkousi, János Nemcsik, Ömür Acet, M. Rosa Bernal-Lopez, Rosa M. Bruno, Rachel E. Climie, Nikolaos Fountoulakis, Emil Fraenkel, Antonios Lazaridis, Petras Navickas, Keith D. Rochfort, Agnė Šatrauskienė, Jūratė Zupkauskienė, Dimitrios Terentes-Printzios

**Affiliations:** ^1^Laboratory for Analytics of Biomolecules, Department of Chemistry in Medicine, Faculty of Medicine, Belgrade, Serbia; ^2^3rd Department of Internal Medicine, Papageorgiou Hospital, Faculty of Medicine, Aristotle University of Thessaloniki, Thessaloniki, Greece; ^3^Department of Family Medicine, Semmelweis University, Budapest, Hungary; ^4^Health Service of ZUGLO, Department of Family Medicine, Budapest, Hungary; ^5^Vocational School of Health Science, Pharmacy Services Program, Tarsus University, Tarsus, Turkey; ^6^Internal Medicine Department, Regional University Hospital of Malaga, Instituto de Investigacion Biomedica de Malaga, University of Malaga, CIBER Fisiopatología de la Obesidad y la Nutrición, Instituto de Salud Carlos III, Málaga, Spain; ^7^Unversite de Paris, INSERM, U970, Paris Cardiovascular Research Center, Paris, France; ^8^Menzies Institute for Medical Research, University of Tasmania, Hobart, TAS, Australia; ^9^Sports Cardiology Lab, Clinical Research Domain, Baker Heart and Diabetes Institute, Melbourne, VIC, Australia; ^10^Faculty of Life Sciences and Medicine, King’s College London - Waterloo Campus, London, United Kingdom; ^11^1st Department of Internal Medicine, University Hospital and Pavol Jozef Šafárik University in Košice, Košice, Slovakia; ^12^Clinic of Cardiac and Vascular Diseases, Institute of Clinical Medicine, Faculty of Medicine, Vilnius University, Vilnius, Lithuania; ^13^School of Nursing, Psychotherapy and Community Health, Dublin City University, Dublin, Ireland; ^14^Centre of Cardiology and Angiology, Vilnius University Hospital Santaros Klinikos, Vilnius, Lithuania; ^15^First Department of Cardiology, Hippokration Hospital, Medical School, National and Kapodistrian University of Athens, Athens, Greece

**Keywords:** vascular aging, circulating biomarkers, oxidative stress, inflammation, cellular matrix, epigenetics

## Abstract

Impairment of the arteries is a product of sustained exposure to various deleterious factors and progresses with time; a phenomenon inherent to vascular aging. Oxidative stress, inflammation, the accumulation of harmful agents in high cardiovascular risk conditions, changes to the extracellular matrix, and/or alterations of the epigenetic modification of molecules, are all vital pathophysiological processes proven to contribute to vascular aging, and also lead to changes in levels of associated circulating molecules. Many of these molecules are consequently recognized as markers of vascular impairment and accelerated vascular aging in clinical and research settings, however, for these molecules to be classified as biomarkers of vascular aging, further criteria must be met. In this paper, we conducted a scoping literature review identifying thirty of the most important, and eight less important, biomarkers of vascular aging. Herein, we overview a selection of the most important molecules connected with the above-mentioned pathological conditions and study their usefulness as circulating biomarkers of vascular aging.

## Introduction

The process of aging is defined as ‘significant physiological alterations which lead to increased susceptibility to disease and risk of death.’ To assess the changes leading to biological aging, molecular and cellular biomarkers, as well as non-invasive imaging techniques, can be applied (Ferrucci et al., 2020). Aging can be viewed as a cell-specific deterioration process mainly caused by genomic instability, telomere shortening, epigenetic changes, and a loss of proteostasis (Crimmins et al., 2008; Freitas-Rodríguez et al., 2017). Moreover, many relevant age-related changes occur at the intercellular level too, which call for a more integrative approach of the aging process (Groenwagen et al., 2016; Sena et al., 2018).

Vascular aging is a natural process, happening with advanced age, and is associated with structural and functional changes in the vascular wall. This is evident in the structural properties of the blood vessels, in that for example, the larger elastic arteries exhibit an increase in collagen content, covalent crosslinking of the collagen, elastin fracture, and calcification and reduction in the elastin content. Increased vascular smooth muscle growth is also a hallmark, in addition to an increase in wall thickness (i.e., the media to lumen ratio). In terms of changes in the functional properties, these are related mainly to aspects of endothelial dysfunction and decreased nitric oxide (NO) production. Finally, and perhaps the major consequence and observable change associated with vascular aging is reduced compliance/increased arterial stiffness of the arterial wall, which is recognized as the hallmark of vascular dysfunction in healthy aging. Importantly, the afore mentioned processes which are associated with vascular aging are accelerated in cardiovascular, and other disease states, and development or onset of these pathophysiological states can result in vascular aging developing earlier in life as compared to natural aging (Nilsson et al., 2008). In this way, as aging is characterized as a chronic progressive proinflammatory phenotype (Franceschi et al., 2000), indirectly controlled by a network of cellular and molecular defense mechanisms, the events which lead to the early development of vascular aging sees many of these mechanisms activated and in turn influence aspects of vascular tree. As such, inflammaging; the gradually adaptive process of the body which leads to a proinflammatory status with advancing age, is central to vascular aging (Kirkwood, 2018).

Another central hallmark and key driver of aging is senescence. Cellular senescence is a state of a durable, irreversible cell-cycle arrest of previously replication-competent cells, which plays a dual role in physiology and disease (Gorgoulis et al., 2019). In this regard, transient induction of senescence followed by tissue remodeling has been recognized as a beneficial mechanism to eliminate damaged or aged cells (He and Sharpless, 2017). Conversely, persistent senescence and inability to eliminate the excess damaged cells, has been linked with detrimental effects leading to inflammation, which as mentioned, is an essential pathophysiological mechanism inextricably linked to tissue dysfunction which characterizes aging and the aging-related diseases (Di Micco et al., 2021).

The term biomarker refers to a broad range of biological measures that can be objectively measured and evaluated as indicators of normal biological and pathogenic processes or pharmacologic responses to a therapeutic intervention (Biomarkers Definitions Working Group, 2001). Specific to aging, or indeed vascular aging, there is currently no standard set of biomarkers in clinical trials yet. The authors of this manuscript were convened within the European Cooperation in Science and Technology (COST) Action on vascular aging international scientific network (CA18216 - Network for Research in Vascular Aging, VascAgeNet^1^, action duration 4 years (November 5, 2019–November 4, 2023) and a comprehensive literature review was conducted specific to this.

In the following, we introduce a selection of identified molecules/cellular aspect which demonstrate potential in addressing the aforementioned deficit, and provide an overview of their usefulness as circulating biomarkers of vascular aging.

## Methodology

In order to identify all relevant studies a literature search was conducted using PubMed. Research articles were also selected manually from the reference lists of articles. The search strategy used the terms “circulating biomarker,” “cardiovascular disease (CVD),” “aging,” “vascular aging,” and the initial selection of biomarkers was refined by those which appeared in most studies, and importantly met the criteria introduced by Vlachopoulos et al. (2015). As a result, Supplementary Table S1 contains the list of extracellular biomarkers which meet these requisites, and are proven to be involved in: inflammation (3), alterations to the cellular matrix (5), aging of the kidney (1), stress response and mitochondria (1), nutrient signaling (1) and epigenetics (3). Supplementary Table S2 contains a subset of miRNA-based biomarkers which have been shown to be involved in influencing genetics (1), the cell matrix (1), inflammation (1), angiogenesis, cell adhesion, and inflammation (1), mitochondria biogenesis and metabolism (2), and metabolism (2). We then further refined this list, ranking the biomarkers based on their influence on: underlying mechanisms related to vascular aging, involvements in disease, proof of concept, prospective validation, incremental value, clinical utility, clinical outcomes, cost-effectiveness, ease of use, methodological consensus and finally reference value (Vlachopoulos et al., 2015). In doing so, we selected only those which aligned closest with the aforementioned criteria. Further refinement of biomarkers was then based on: (1) clinical utility; (2) cost effectiveness; (3) ease of use; (4) methodological consensus; and (5) reference values (cut-off values).

In conducting the literature search, duplicated studies were identified and removed. The abstracts and titles of article retrieved were screened to exclude the irrelevant studies. Full-text articles were then examined to determine whether they met the inclusion criteria. Inclusion criteria were: (1) studies investigating the association of a biomarker with CVDs, and vascular aging; (2) studies using blood serum or plasma for biomarker analysis; and (3) peer-reviewed articles and all types of reviews published in English between January 1989 and October 2021. Unpublished theses, reports, and conference proceedings were excluded. Data were extracted using a standardized form by the reviewers and verified by another reviewers.

We aim to address the vascular aging phenomenon of different factors within the scope of COST 18216 “Network for Research in Vascular Aging” action. For each selected biomarker, we present the data according to four different sections: pathophysiology, association with physiological aging and vascular markers, association with CVD and measurement method. This review describes important molecules associated with pathological conditions and their implications for their usefulness as biomarkers of circulating vascular aging and showing relevant data on outcomes whenever available. Reviews, guidelines, and consensus papers are also included.

## Results

Thirty of the most important and pertinent biomarkers related to inflammation, matrix injury, stress response and mitochondria, nutrient signaling, vascular senescence, thrombotic events and epigenetic were identified; and eight of the less important biomarkers related to genetics, matrix remodeling, angiogenesis, cell inflammation, mitochondrial biogenesis, metabolism (Supplementary Tables S1, S2).

**FIGURE 1:**
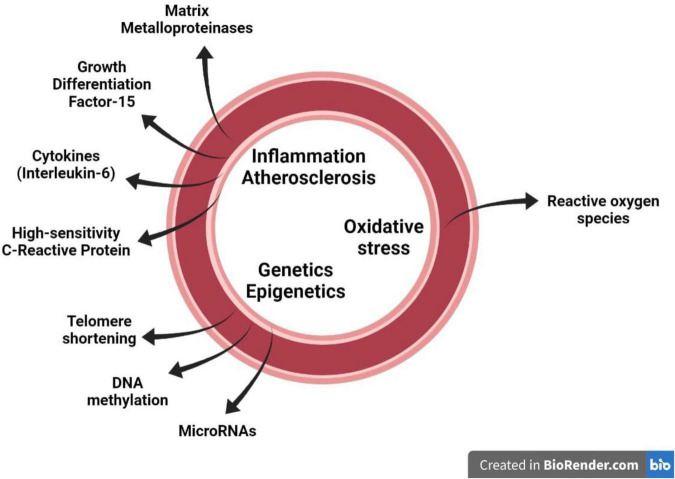
Several circulating biomarkers which are proposed to mirror the major pathophysiological mechanisms that contribute to vascular aging in the vascular wall, namely inflammation/atherosclerosis, oxidative stress and genetics-epigenetics.

Application of stated criteria to the available literature to the identified 38 biomarkers resulted in a short list of circulating biomarkers: oxidative stress based – superoxide dismutase (SOD); inflammation based – high sensitivity C-reactive protein (hsCRP) and interleukin-6 (IL-6); cellular matrix based – matrix metalloproteinases (MMPs), growth differentiation factor-15 (GDF-15); epigenetic based – micro ribonucleic acids (miRNAs), DNA methylation and telomere length (Figure 1).

## Circulating Biomarkers Related to Vascular Aging

### Oxidative Stress-Based Circulating Biomarkers

#### Reactive Oxygen and Nitrogen Species

##### Pathophysiology

Reactive oxygen and nitrogen species (RONS) are formed by the use of molecular oxygen by aerobic organisms and play an essential role in physiology and pathophysiology of aerobic life (Li et al., 2016). RONS are a group of small reactive molecules, including radicals and non-radicals, that are derived from oxygen metabolism. RONS with unpaired electrons are considered as free radicals. Free radicals such as superoxide ion (${\text{O}}_{2}^{∙-}$) and hydroxyl radical (OH^∙⁣–^), are unstable in nature and subsequently have short biological half-lives (Stevenson et al., 2020). Non-radicals in comparison; such as hydrogen peroxide (H_2_O_2_), singlet oxygen (^1^O_2_), peroxynitrite (ONOO^–^) and hypochlorous acid (HOCl), are more stable in contrast and thus exhibit longer half-lives as compared to their free radical counterparts. Mitochondrial respiration is a major source of reactive oxygen species (ROS) within most cells. As electrons are transferred between the complexes of the electron transport chain, some of these electrons can ‘leak’ out to react directly with oxygen to form superoxide (Shields et al., 2021). A hallmark of aging is the progressive loss of mitochondrial function and in turn reduction in mitochondrial-derived ROS (Santos et al., 2018).

In that way, whilst aging sees a reduction in mitochondrial levels of ROS, with respect to the vascular system, there are several other sources of RONS which play a role in promoting oxidative stress; most notably; the enzymatic sources of RONS; which have been the subject of extensive investigation the last number of years. Notably, NADPH oxidases (NOX); which utilizes electrons donated from NADPH to convert molecular oxygen to ${\text{O}}_{2}^{∙-}$, myeloperoxidases (MPO); which converts H_2_O_2_ to HOCl, xanthine oxidase (XO); which produces ${\text{O}}_{2}^{∙-}$ anions during the breakdown of purines to uric acid, SOD; which converts ${\text{O}}_{2}^{∙-}$ anions to H_2_O_2_ and oxygen, and monoamine oxidase; which decomposes dopamine and produces H_2_O_2_ (Shields et al., 2021), are among the most prominent sources of vascular ROS, closely followed by lipoxygenase (LOX), cyclooxygenase (COX), angiotensin II, and cytochrome P450 1B1.

According to numerous published data, RONS have a dual role in an organism: RONS contribute to aging but they also play a crucial role in cell signaling and development, thus serving a beneficial role. In the vascular system, physiological levels of RONS are essential for normal vascular functions including endothelial homeostasis and smooth muscle cell contraction (Salisbury and Bronas, 2015). For example, the free radical nitric oxide (NO), which is produced by endothelial nitric oxide synthase (eNOS) by the vascular endothelium, is very important in the regulation of blood flow and vasodilation (Chen et al., 2018). However, any imbalance between prooxidant and antioxidant mechanisms, often called “redox potential,” can play an important role in vascular aging (Ferrini et al., 2004). Excessive levels of ROS have been shown to cause vascular remodeling by inducing proliferation and migration of vascular smooth muscle cells, vascular cell damage, the recruitment of inflammatory cells, lipid peroxidation, activation of metalloproteinases and deposition of extracellular matrix (Chen et al., 2018). The initiation and progression of disease states such as hypertension, atherosclerosis, restenosis and abdominal aortic aneurysm are among the main vascular diseases in which oxidative stress has been shown to play a key role (Tejero et al., 2019).

With respect to reactive nitrogen species (RNS), a large proportion of their production stems from the activity of eNOS. Under pathological conditions, eNOS can also produce ${\text{O}}_{2}^{∙-}$ which contributes directly to ROS levels. The ${\text{O}}_{2}^{∙-}$ can now react with the NO produced by the same enzyme to produce peroxynitrite (ONOO^–^), which has been shown to directly damage cellular components, or further react with other molecules to create other types of RNS; nitric dioxide (NO_2_^∙^), nitrate radical (NO_3_^∙^), nitrous acid (HNO_2_), nitrite (NO_2_^–^), nitrosyl cation (NO^+^), nitroxyl anion (NO^–^), peroxynitrous acid (ONOOH), dinitrogen trioxide (N_2_O_3_). Similar to peroxynitrite, these RNS have also been shown to cause similar nitrosative damage to cellular component, but also have functional roles in cell signaling and pathogen defense (Shields et al., 2021).

Excessive levels of RONS are mainly generated by and credited to the NOX family of enzymes. The NOX family are the only family of enzymes which produce RONS as its primary function (Lassegue et al., 2012; Montezano and Touyz, 2014), and in general NOX-derived ROS are important regulators of endothelial function and vascular tone. However, the excessive reduction of molecular oxygen to superoxide anion ${\text{O}}_{2}^{∙-}$ by members of this family has been linked to vascular disease and biological aging, directly and indirectly, via several pathogenic pathways (Blankenberg et al., 2003). For example, ${\text{O}}_{2}^{∙-}$ from NOX enzymes initiates the process of eNOS uncoupling which promotes accelerated vascular aging in CVD. Moreover, SOD dismutates the superoxide anion to H_2_O_2_ which is able to form highly reactive hydroxyl ions (OH^∙^) (Gong et al., 2014), that are extremely reactive and cause damage to the cell membrane phospholipids and proteins (Liguori et al., 2018). All seven family members of the NOX family are major producers of RONS in mammalian cells, yet NOX-1, -2. -3, -4, and -5 are variably expressed in the vascular system by endothelial cells or vascular smooth muscle cells, and are among those identified as having a predominant role, through their specific generation of ${\text{O}}_{2}^{∙-}$ or H_2_O_2_, in progressing vascular disease (Ighodaro and Akinloye, 2018). Of note, the exact pathophysiological significance of NOX-5 is still unclear, but according to the review of Touyz et al. (2019), this enzyme is important in the physiological regulation of sperm motility, lymphocyte differentiation and interestingly vascular contraction. Emerging evidence has implicated its hyperactivation in the onset and development of CVD, as well as kidney injury and cancer.

Furthermore, in fully understanding the redox dynamics central to pathophysiological disease states, attention has been directed toward the inherent physiological defense mechanisms which protect the body against oxidative stress. The most important enzymatic systems include SOD, catalase (CAT), glutathione peroxidase (GPx), glutaredoxin (Grx), thioredoxin (Trx), peroxiredoxin (Prx) and glutathione S-transferase (GST). These antioxidant enzymes catalytically remove ROS in their own unique way; for example, SOD dismutases ${\text{O}}_{2}^{∙-}$ into H_2_O_2_, which is in turn degraded by CAT or GPx into water and molecular oxygen. Grx and Trx act to protect thiol-containing proteins by repairing damage caused by ROS by transferring electrons to the disulfide bonds of the damaged proteins in order to repair them and restore them back to its reduced form. Prx is a cysteine containing enzyme that reduces the activity of peroxides, while GSTs act synergistically with GPx to reduce lipid peroxidation.

Among the non-enzymatic antioxidants, the most pertinent is glutathione (GSH); which is essential for the activity of GPx and Grx in returning each to their active state. *N*-Acetyl cysteine (NAC) is important in the formation of GSH acting as its precursor molecule, whilst also participating in redox reactions by donating electrons toward the detoxification of ROS which are present in the vicinity. Finally, Vitamin C and E are important water-soluble antioxidants which scavenge oxygen free radicals in the environment and prevent the oxidation of cholesterol. Vitamin E also acts as an inhibitor of lipid peroxidation (Nandi et al., 2019; Shields et al., 2021).

##### Association With physiological Aging and Vascular Markers

One of the main features of aging is a progressive loss of tissue and organ function over time. In accordance with the free radical theory of aging; age-associated functional losses are due to the accumulation of oxidative damage to macromolecules (lipids, DNA, and proteins) caused by RONS. The exact mechanism of aging caused by oxidative stress is still unclear, but oxidative stress and aging are becoming increasingly recognized as intertwined biological events as increased RONS production is observed with aging. A wealth of experimental data confirms oxidative stress disrupts critical signaling pathways owing to the damage inflicted to the aforementioned macromolecular molecules/structures of cells and tissues. These changes in signaling pathways are correlative to those associated with cellular aging; a physiological mechanism that reduces/halts cell proliferation in response to damage incurred during the replication cycle (Papaconstantinou, 2019). Senescent cells acquire an irreversible senescence-associated secretory phenotype (SASP) involving secretion of soluble factors (interleukins, chemokines, and growth factors), degradative enzymes like MMPs, and insoluble proteins/extracellular matrix (ECM) components. For example, adoption of a senescent phenotype in endothelial cells leads to impaired endothelium-dependent vasodilation and upregulation of inflammatory gene expression in aged vasculature (Donato et al., 2015). This data and others, have implicated RONS as inducers of cellular senescence in directly acting on various components associated with SASP (Liguori et al., 2018).

Thereafter, RONS-mediated events are concurrent with vascular inflammation and the development and progression of atherosclerosis and accelerated atherosclerotic damage (Wu et al., 2014; Kattoor et al., 2017). Recent experimental data have linked RONS production with the development of aortic stiffening, while conversely, indirect clinical evidence supports the beneficial effect of antioxidant therapies in reducing arterial stiffness (Ashor et al., 2014; Canugovi et al., 2019).

##### Association With Cardiovascular Disease

Substantial evidence clearly shows that oxidative stress, through inflammation, endothelial dysfunction, and other pro-atherosclerotic mechanisms, plays a pivotal role in the pathophysiology of CVD and highly cardiovascular (CV) risk disorders including hypertension, dyslipidemia, peripheral artery disease, metabolic syndrome, and diabetes mellitus (DM) (Loperena and Harrison, 2017; Touyz and Delles, 2019). Of note, several studies have suggested that therapeutic interventions using antioxidants have prevented CV progression. For example, supplementation of Vitamin A, C, and E have demonstrated positive effects in short-term secondary CV prevention, though their exact role in vascular health remains divisive (Ashor et al., 2016). Other trials aimed at addressing the redox balance in individuals are perceived in much the same way; caloric restriction (reduced CV aging and increased chronic disease protection), nutraceuticals (reduction of blood pressure and oxidative stress, with concomitant increases in NO release), and dietary supplements such as fish oil and green tea (improvement in endothelial function, reduction in inflammation and oxidative stress). Moreover, endogenous forms of antioxidant enzymes are now available (SOD, CAT, and Gpx), whilst efforts are ongoing to produce other mimetics or scavenging molecules of similar capabilities, though no molecule has conclusively been successful, while others are still in trials (Izzo et al., 2021). Similarly, direct evidence linking excessive RONS production to CV mortality is currently lacking due to the complexity in the pathophysiology of CVD, hence oxidative stress has been rendered a key contributing factor but not the primary pathogenetic mechanism. Despite these complexities, clinical data from several trials investigating classical antioxidant treatments, but not medications possessing indirect antioxidant properties, have yet to present conclusive evidence of either no benefit or even harm (Senoner and Dichtl, 2019).

It is worth to mention that many studies have suggested the possible therapeutic application of antioxidants. Among antioxidants, vitamin E and C have shown some effect in short term secondary CV prevention, but their role in vascular health remains controversial (Ashor et al., 2016; Izzo et al., 2016). Even more controversial is the supplementation with vitamin A, which even worsened all-cause mortality (Izzo et al., 2021). In some trials antiaging and antioxidant strategies have been documented. They include: caloric restriction (CV aging and chronic disease protection) (Ashor et al., 2016); nutraceuticals (reduction of blood pressure and oxidative stress, increase of NO release; Ashor et al., 2016); dietary supplements such as: fish oil, green tea, vitamin C and A (improvement of endothelial function, reduction of inflammation and oxidative stress) (Ashor et al., 2016). Until now it is impossible to use endogenous antioxidant enzymes, SOD, catalase or GPx, or any synthetic deriving molecule. Several attempts have been made to create such molecules or scavenging enzymes, but so far, no elements have been successful, and other molecules are still in trials (Izzo et al., 2021).

One of the new molecules emerging as important mediators regulating oxidation and inflammation in the vasculature and heart is osteoprotegerin, which may act through reduction in apoptosis and preservation of the matrix structure. Although osteoprotegerin can affect vascular function, its cardiac effects seem to be direct and independent of effects on the vasculature (Guzik and Touyz, 2017). Finding new molecules and new ways to address aging and oxidative stress is fundamental to find new potential application for cardiovascular prevention and treatment.

##### Measurement of Reactive and Nitrogen Oxygen Species

Owing to their extremely short half-lives, it is currently incredibly difficult to accurately measure and assess RONS levels (Rice et al., 2012). Markers of oxidative stress are either molecules modified by interactions with ROS, or molecules of the antioxidant system changed in response to increased redox stress (Tanguy et al., 2000). In that way, targeting the molecules upstream of RONS represents the current best way to assess RONS levels indirectly, and the most commonly used markers in CV research include isoprostanes (IsoPs), malondialdehyde (MDA), nitrotyrosine, *S*-glutathionylation, MPO, oxidized LDL, ROS induced changes to gene expression, glutathione peroxidase (GPX), and isoforms of SOD (Zarzuelo et al., 2013). The measurement of SOD is perhaps one of the most accepted means of measuring RONS activity owing to their levels reflecting their activity catalyzing the dismutation of the strong superoxide radical (${\text{O}}_{2}^{-}$) thus maintaining redox balance and preventing accumulation of injurious ROS (He et al., 2009). There are three distinct SOD isoforms which include the Cu/Zn superoxide dismutase (SOD1), MnSOD (SOD2), and ECM superoxide dismutase (ECM-SOD/SOD3), and as these isoforms remain stable even when the erythrocytes of the samples are hemolyzed and stored frozen (Pierce et al., 2011), they are suggested to be a suitable biomarker for estimation of oxidative stress (Thangaswamy et al., 2012).

### Inflammation-Based Circulating Biomarkers

Nowadays, it is strongly believed that aging and age-associated diseases share many common physiologic and pathophysiologic pathways which seem to converge on inflammation. In fact, at the cellular level, aging is characterized by a state of chronic, sterile, low-grade inflammation (i.e., inflammaging), and it is considered a pre-status mechanism for many age-associated diseases such as cancer, diabetes mellitus (DM) and CVD (Franceschi et al., 2020). Based on the study of Franceschi et al. (2000), inflammaging is the expansion of the network theory of aging and the remodeling theory of aging (Franceschi, 1989; Franceschi and Cossarizza, 1995). Based on the remodeling theory, inflammaging is the gradual adaptive process of the body; the net result of which is a regulation of malignant damage sustained by the body as a result of a trade-off with the immune system. Despite the lack of agreement on definitions and terminology surrounding inflammaging, the prevailing consensus is that the primary feature of such is an increase in the proinflammatory status with advancing age (Kirkwood, 2018).

From an evolutionary perspective, inflammaging is primarily driven by several endogenous signals including the accumulation of misplaced and misfolded self-molecules from damaged or senescent cells. Among the several inflammatory factors, the key molecules associated with inflammaging and aging are the elevated pro-inflammatory cytokines, especially interleukin (IL)-6 and C-reactive protein (CRP) (Singh and Newman, 2011). Various studies have described production of IL-6 by endothelial cells. More specifically, it has been described that human umbilical vein endothelial cells (HUVECs) can produce IL-6 via a G-protein, calcium, and NF-kB-dependent pathway upon stimulation of the protease-activated receptors (PAR) PAR-1 or PAR-2. The effects of both PAR-1 and PAR-2 agonists on the endothelial cells are greatly enhanced by concomitant stimulation by endotoxin (lipopolysaccharide, LPS) or tumor necrosis factor-a (TNF-a) (Chi et al., 2001). In addition, in acute inflammation, it has been shown that thrombin (a procoagulant and proinflammatory molecule) may induce monocyte recruitment through endothelial activation by inducing monocyte chemotactic protein-1 (MCP-1) secretion indirectly through an autocrine loop involving endothelial IL-6 secretion (Marin et al., 2001). Bacterial endotoxin or inflammatory cytokines, such as IL-1 or TNF-alpha have also been shown to stimulate IL-6 production in adult vascular endothelial cells (Loppnow and Libby, 1989; Naka et al., 2002; Puel and Casanova, 2019).

#### Interleukin-6

##### Pathophysiology

Interleukin-6 is a small, 21 kDa glycoprotein produced by numerous cell types, including but not limited to; dendritic cells, macrophages, monocytes, T cells and vascular cells. IL-6 binds to its receptor (IL-6R), thus activating the Janus kinase (JAK) tyrosine kinases and the downstream signal transducer and activator of transcription (STAT). The net result is a pleiotropic tissue activity predominantly directed toward the initiation of inflammation and the elevation of acute phase reactants including CRP (Hirano and Murakami, 2020).

##### Association With Physiological Aging and Vascular Markers

Based on current knowledge, IL-6 is a well-acknowledged sensitive biomarker of low-grade inflammation and is among the best candidates within the interleukin family as a representative biomarker of vascular aging (Puzianowska-Kuźnicka et al., 2016). IL-6 has been positively associated with vascular markers of aging such as pulse wave velocity (PWV) in healthy men and patients with chronic kidney disease (Nishida et al., 2007; Peyster et al., 2017), and carotid intima-media thickness (cIMT) (Huang et al., 2016). Additionally, IL-6 levels have been found to increase in an age-dependent manner in two cohorts of older individuals (Puzianowska-Kuźnicka et al., 2016; Stevenson et al., 2018). Similarly, in a recent meta-analysis including 12421 values for IL-6 in the blood of healthy adult male and female donors, it was found that for every 1-year increase in age, there was a significant increase of IL-6 values by 0.05 pg/ml (Said et al., 2021). Moreover, there is substantial evidence that IL-6 contributes to hypertension as levels of IL-6 have been correlated with blood pressure in hypertensive subjects and are reduced by treatment with angiotensin II-receptor blockade (Vázquez-Oliva et al., 2005). A similar study corroborates this relationship in demonstrating that angiotensin II-receptor inhibitor significantly reduces IL-6 levels in hypertensive patients (Tsutamoto et al., 2000; Manabe et al., 2005).

Hypertension and atherosclerosis are associated with accumulation of cellular senescence biomarkers in the vascular wall; a hallmark often associated with vascular dysfunction (Kovacic et al., 2011). Additional risk factors for CVD; such as smoking, hyperlipidemia, or DM, are also associated with accelerated decline of vascular function (Cunha et al., 2017). Hypertension is inherently associated with accelerated vascular aging and, therefore, research on such has assisted in understanding the vascular remodeling that occurs with age (Lakatta, 2007; Huang et al., 2018). Early vascular aging—a term introduced in the context of premature development of vascular stiffness and remodeling by Nilsson et al. (2009) and Cunha et al. (2017) is a key feature of hypertension (Lakatta, 2007; Guzik and Touyz, 2017). In hypertensive patients, the vascular wall activates monocytes, which increases the release of IL-6 (Guzik and Touyz, 2017; Loperena et al., 2018). Concurrently, the existence of a chronic inflammatory state has been reported in elderly age, and has been shown to be caused by an increase in the levels of IL-6 and TNF-alpha (Derhovanessian et al., 2009). In that way, recent advances in hypertension research have unraveled novel inflammatory, and oxidative mechanisms, of vascular dysfunction that underlie accelerated vascular aging in hypertension and associated CVD (Passacquale et al., 2016).

##### Association With Cardiovascular Disease

A wealth of data exists confirming the association between increased IL-6 levels and CV mortality and morbidity (Libby et al., 2002; Rao et al., 2005; Danesh et al., 2008; Compté et al., 2013; Ridker et al., 2018; Batra et al., 2021).

More specifically, in the population-based Cardiovascular Health Study, elevated plasma IL-6 levels were found to significantly associate with death across multiple causes and strongly predict future mortality (Walston et al., 2009). Similarly, higher IL-6 levels were associated with increased mortality in older adults in the Framingham Heart Study (Roubenoff, 2003). More recently, it was demonstrated that for each SD increase in log IL-6, there was a 25% increase in the risk of future CV events (Kaptoge et al., 2014). Several studies described the association of IL-6 and cardiovascular mortality and morbidity (Held et al., 2017; Wainstein et al., 2017; Ridker et al., 2018).

##### Measurement of Interleukin-6

Kenis et al. (2002) examined the stability of IL-6 in human serum using an accelerated stability testing protocol according to the Arrhenius equation. In this study, the effect of time delay between blood sampling and sample processing, clotting temperature and repeated freeze-thaw cycles on serum levels of these proteins were determined. It was concluded that serum samples for the determination of IL-6 can be stored at −20°C for several years (Kenis et al., 2002). Graham et al. (2017) studied the impact of initial and multiple subsequent freeze-thaw cycles on pro-inflammatory including IL-6, anti-inflammatory, acute phase proteins and other biomarkers. The authors found that the examined biomarkers on their panel remained stable for analysis despite multiple freeze-thaw cycles. Together, these data provide the foundation and confidence for large scale analyses of panels of inflammatory biomarkers to provide better understanding of immunological mechanisms underlying health versus disease (Graham et al., 2017).

The foremost way of measuring IL-6 levels in a biological sample is by enzyme-linked immunosorbent assays (ELISA) which are readily available from numerous companies today. For this analysis, sample volumes as little as 50 μL are measurable, with sample matrices including but not limited to serum, plasma, cell culture supernatant. Most ELISA formats allow for a measurable range of 1.56–100 pg/mL (Tecan manufacturer of ELISA kits). Owing to the nature of ELISA-based methods, results of the study Gong et al. (2019), highlighted a number of variables which can affect levels of cytokine in a sample. It was found that incorrect sample handling procedures played a key role in the obtained results. For example, plasma and serum sampling require their own individual protocols. With respect to IL-6, unseparated EDTA plasma can keep IL-6 stable for up to 24 h, whereas with unseparated serum it is recommended to measure IL-6 as soon as possible. Despite these potential obstacles, ELISAs have improved upon earlier methods which utilized flow cytometry and determined the levels of IL-6 based on the cellular response to the cytokine and the employment of a dose-response curve. In comparison, levels of cytokines like IL-6 and their respective activity can now be rapidly determined in accordance with the World Health Organization (WHO) cytokine standards; taking as little as a few hours to achieve, in comparison to previous methods which took days in comparison (Simard et al., 2014).

#### High Sensitivity-C-Reactive Protein

##### Pathophysiology

C-reactive protein or high sensitivity (hs)-CRP is an acute-phase reactant and a nonspecific inflammatory biomarker whose production is stimulated in response to inflammatory mediators including IL-6 and IL-1. It is predominantly secreted by hepatocytes in the form of pentameric molecules, although low-level expression of CRP in other cells has also been observed. Levels of CRP have been shown to increase rapidly up to 1,000-fold at sites of trauma, infection or inflammation, and accordingly, rapidly decrease upon resolution of the causative condition. Measurement of hs-CRP is therefore widely used to monitor various inflammatory states.

##### Association With Physiological Aging and Vascular Markers

Increasing evidence shows that hs-CRP is not only an inflammatory biomarker but also an important risk factor associated with aging and aging-related diseases (Tang et al., 2017). Pertinent to this, it has been demonstrated that hs-CRP levels increase in an age-dependent manner across aging elderly populations without evident CVD. Relatively, hs-CRP levels are significantly lower in healthy aging adults compared to those with aging-related diseases (Puzianowska-Kuźnicka et al., 2016). Moreover, high hs-CRP levels are associated with decreased physical and cognitive performance which are strongly related to the natural aging process (Puzianowska-Kuźnicka et al., 2016; Tegeler et al., 2016), and, additionally, with several aging diseases like sarcopenia (Lee et al., 2020), deep white matter lesions, ischemic stroke, and heart failure (van Wezenbeek et al., 2018).

Concerning the association of hs-CRP with vascular disease, a connection between hs-CRP and atherosclerosis has long been established since hs-CRP directly binds oxidized low-density lipoprotein cholesterol (LDL-C) and has been shown to be present within atherosclerotic plaques. As a result, hs-CRP contributes to plaque instability and exerts a highly proatherogenic effect (Singh et al., 2008; Conte E. et al., 2020). Moreover, hs-CRP has been associated with indices of vascular stiffness. Relatively, hs-CRP has been found to positively correlate and, additionally, predict elevated PWV over and above traditional CV risk factors and in various diseased populations (Nakhai-Pour et al., 2007; Mozos et al., 2019). In addition, hs-CRP has been positively correlated with markers of carotid stiffness including cIMT (Liao et al., 2014).

##### Association With Cardiovascular Disease

The association between elevated hs-CRP levels and CVD is well established since most large-scale clinical trials have used a hs-CRP cut off point of 2 mg/l for determining increased CV risk, and a handful of studies have shown a consistent association of hs-CRP levels above 3 mg/l with CV events (Möhlenkamp et al., 2011). Additionally, it has been demonstrated that hs-CRP levels are able to predict future CV events. In a large meta-analysis of 160,309 individuals without a history of vascular disease, among a total of 27,769 patients who suffered fatal or nonfatal events, hs-CRP was associated with a significantly increased risk for coronary heart disease (CHD), stroke, and vascular mortality (Kaptoge et al., 2009). Similarly, ample studies have confirmed that elevated hs-CRP concentrations can independently predict the risk of all-cause and CV mortality in different populations including the general population and patients with CHD (Li et al., 2017; Stumpf et al., 2017).

Due to its strong association with CVD, many mathematical models have tried to incorporate hs-CRP in order to improve CV risk prediction owing to the high correlation of hs-CRP with multiple risk factors. Subsequently, hs-CRP has been included in the position paper of the European Society of Cardiology and the Artery Society (Vlachopoulos et al., 2015), and it has been integrated into numerous guidelines in different pathological CV conditions (for primary and secondary CVD prevention). However, the class of recommendations for hs-CRP as circulating biomarker related to vascular wall biology may be measured as part of refined risk assessment only in patients with an unusual or moderate risk, whose profile is mostly class IIb with a level of evidence B, but not in asymptomatic low-risk or high-risk individuals (class III and level of evidence B recommendation) (Perk et al., 2012; Vlachopoulos et al., 2015). Therefore, there seems to be a modest support of the incremental value of hs-CRP, and it is debatable whether its measurement can provide a consistent and clinically meaningful incremental predictive value in risk prediction and reclassification (Yousuf et al., 2013).

##### Measurement of High Sensitivity-C-Reactive Protein

The standard CRP turbidimetric immunoassay measures markedly high levels of the protein in the range from 10 to 1000 mg/L. On the contrary, the hs-CRP assay accurately detects even lower, basal levels of CRP in the range from 0.5 to 10 mg/L which belongs to the currently accepted CV risk assessment range of 0.20–10.0 mg/l.

### Cell Matrix Based Circulating Biomarkers

#### Matrix Metalloproteinases

##### Pathophysiology

Proteases degrade proteins by hydrolyzing their peptide bonds, and in their action control and influence many key physiological and pathological processes (López-Otín and Hunter, 2010). An essential part of each cell; the ECM can be modified during aging by protease dysfunction. Imbalances or altered activity of certain proteases can ultimately affect the structure and composition of the ECM, thus altering its ability to perform its biological functions; differentiation, proliferation, migration and survival (Freitas-Rodríguez et al., 2017). Matrix MMPs are a family of multidomain calcium-dependent, zinc-containing endopeptidases, activated by inflammatory signaling. They are able to degrade ECM molecules, have been shown to have a crucial role in aspects of aging, hypertension and atherosclerosis within the arterial wall (Wang et al., 2014). Activated MMPs influence arterial remodeling by promoting endothelial inflammation, intimal-medial thickening, elastin fiber network destruction, arterial fibrosis, calcification and adventitial expansion. Importantly, it was shown that aging enhances MMP-2/-7/-9/-14 activity in the aortic wall, namely via increases in Ang II-mediated signaling, proinflammation, fibrosis and elastin fragmentation (Wang et al., 2015). Conversely, human tissue inhibitors of matrix metalloproteinases (TIMPs) are four glycoproteins responsible for the inhibition of MMPs and thereby are also involved in and influence degradation of ECM.

##### Association With Physiological Aging and Vascular Markers

There is significant discordance between current and previous studies regarding the association of MMPs with age. For example, in a study including 93 healthy adults of different ethnic origins, no associations were found between MMP-2 and MMP-9 with age (Tayebjee et al., 2005), nor any gender influence. Similarly, in 699 adults of the Framingham study, MMP-9 was not associated with age (Sundström et al., 2004). In a subsequent study including 77 subjects with no evidence of CVD, significant positive correlations between MMP-2 and MMP-7, and a significant negative correlation between MMP-9 and age were found (Bonnema et al., 2007). Recently, Basisty et al. (2020), in using proteomic technology, identified increased levels of MMP-1, MMP-2 and their regulators TIMP1 and TIMP2 as secreted by senescent cells in human plasma (Basisty et al., 2020). Interestingly, age-related changes in the cardiac proteome have been also shown to be MMP-9 dependent (Iyer et al., 2016), while age-related MMP-2 upregulation occurs in the human aorta (McNulty et al., 2005).

Taking into account the crucial role of MMPs into vascular pathophysiology, a certain link with vascular function markers is anticipated. Indeed, it has been shown that plasma MMP-1 levels display a positive correlation with both PWV and augmentation index (AIx) after adjustment for age and mean arterial pressure in a cohort of normotensive and hypertensive individuals (McNulty et al., 2005). Similarly, MMP-9 levels have been positively correlated with aortic and brachial PWV in patients with hypertension including isolated systolic hypertension (Yasmin et al., 2005; Tan et al., 2007; Gkaliagkousi et al., 2012), whereas MMP-2 and MMP-3 have been associated with central arterial stiffness parameters in patients with DM type I (Peeters et al., 2017). In the most recent study so far including 206 healthy young adults, MMP-3 levels were significantly associated and independently predicted PWV (Iannarelli et al., 2021).

##### Association With Cardiovascular Disease

Subsequently, sample data confirm that several MMPs are closely associated with CVD and can predict both CV events and all-cause mortality in various populations including patients with DM, atherosclerosis and the general population (Table 1).

**TABLE 1:** Extracellular matrix biomarkers as predictors of risk in CVD.

Biomarker	Tissue	Clinical context	No. of subjects/type of the study/length of follow-up	Main findings	References
*MMP-1*	Serum	Atherosclerosis	260/cross-sectional study/–	Serum levels associated with plaque burden	Lehrke et al., 2009
*MMP-2*	Plasma	Type 1 diabetes	337/cohort/12.3	Association with CVD	Peeters et al., 2017
*MMP-8*	Serum	CAD, MI	7928/cohort/13	Predictor of mortality	Kormi et al., 2017
*MMP-9*	Plasma Serum Serum	CVD MI, Stroke CVD	1127/cohort/4.1 1082/cohort/18.1 922/Framingham/9.9	Predictor of cardiovascular mortality Relevant marker of CV mortality Association with mortality	Blakenberg et al., 2003 Hansson et al., 2011 Velagaleti et al., 2010
*MMP-10*	Serum	PAD	187/cohort/2	Increased levels associated with mortality	Martinez-Aguilar et al., 2015
*MMP-12*	Plasma	Atherosclerosis, CAD, T2DM	1500/proximity extension assay technology/–	Plaque development	Goncalves et al., 2015
*TIMP-1*	Plasma	CVD	389/prospective/2	Predictive of all-cause death, MI and cardiac mortality	Cavusoglu et al., 2006

*CAD, coronary artery disease; CVD, cardiovascular disease; PAD, peripheral artery disease; MI, myocardial infarction; T2DM, type 2 diabetes mellitus; –, no data.*

##### Measurement of Matrix Metalloproteinases

Determination of MMPs in biological samples (primarily serum or plasma) can be performed by several analytical methods: ELISA, zymography; optical methods such as near-infrared (IR) optical imaging, fluorescence, and surface plasmon resonance spectroscopy; the use of active-site probes followed by enzymatic digestion of the captured MMPs, and in the last two decades liquid chromatography-mass spectroscopy (LC-MS/MS) (Lopez-Avila et al., 2008). Gelatin zymography is widely used for the detection of the gelatinases MMP-2 and MMP-9 at a level of 10 pg (Kleiner and Stetler-Stevenson, 1994). Casein-, collagen-, and heparin-enhanced substrate- zymography are suitable for the higher quantities of other members of MMPs, while reverse zymography can be used for the detection of TIMPs (Tunon et al., 2010; Hoefer et al., 2015).

Most of the aforementioned assays for MMPs rely on the biological activity of the enzyme to degrade natural substrates, and are so-called “bioassays.” A basic problem in utilizing bioassays in general is that they lack specificity against different substrates, however, they are very sensitive in their level of detection [MMP-9 – 12.5 ng (1.0 nM), MMP-2 – 20 ng (2 nM)]. Given there are a multitude of ways to measure MMPs, each approach presents with its own distinct advantages and disadvantages. For example, zymography as mentioned is perhaps the most routine approach for the detection of particular MMPs based on their activity relative to a relevant substrate, however, a disadvantage of the technique lies in the influence different anticoagulants can have as pre-analytical determinants of plasma MMP activities (this approach should only be used on plasma samples collected in heparin-free collection tubes). In comparison, *in situ* zymography utilizes an antibody raised to target specific MMP species, which upon interaction is captured and immobilized in areas where MMPs are present before subsequently being utilized for detection and quantification processes. This method is quick, and demonstrates good sensitivity for each subtype of MMP as compared to zymography and collagenolysis. However, this mode of analysis has been shown to possibly influence physical tissue parameters of samples. Western blotting (protein blotting or immunoblotting) is a powerful and important procedure for the immunodetection of MMPs post-electrophoresis. Presenting some unique advantages; in that the resultant membranes are pliable and easy to handle, and the proteins which are immobilized on the membrane are readily and equally accessible to different ligands, Western blotting is utilizes antibodies in much the same way as *in situ* zymography does, in being specific to the MMP of interest but is perhaps more versatile in its approach. However, Western Blotting does require knowledge of the MMPs native substrate and the availability of anti-MMPs antibodies. In this way, this approach can be expensive and time-consuming. Owing to these respective limitations, ELISAs have emerged as a popular approach for measuring the abundance of MMPs in a sample, and currently ELISAs are available for a number of MMPs species; MMP-9 and MMP-2 and their specific tissue inhibitors: TIMP-1 and TIMP-2 ELISA kits are commercially available and have a good sensitivity (Cheng et al., 2008).

### Growth Differentiation Factor 15

#### Pathophysiology

Growth differentiation factor 15 is a member of the transforming growth factor-β (TGF-β) cytokine superfamily. Also known as macrophage inhibitory cytokine 1 (MIC-1), under physiological conditions it is found in abundance only in placenta (Fairlie et al., 1999). However, as an autocrine regulator of macrophage activation, its levels increase upon macrophage activation and it is produced under conditions of inflammation, vascular injury, and oxidative stress from human endothelial and vascular smooth muscle cells (Bootcov et al., 1997). Its role in vascular biology is divergent as evidence suggests it can exert either pro- or anti- inflammatory, angiogenetic and apoptotic effects (Unsicker et al., 2013). In this context, GDF-15 is released by macrophages during acute phase responses, and its level is thus influenced by pro-inflammatory cytokines such as IL-1, TNFα, TGF-β and CRP.

#### Association With Physiological Aging and Vascular Markers

Growth differentiation factor 15 is a molecule directly linked to inflammaging. Recently, in a study investigating the plasma proteomic signature of aging in healthy humans, it was demonstrated that GDF-15 levels rise with aging, and exhibit the strongest positive association with age. Interestingly, in the same study, GDF-15 levels were not associated with other CV risk factors indicating that GDF-15 may specifically represent a biomarker of aging (Tanaka et al., 2018). In addition, it has been shown that GDF-15 is produced by senescent endothelial cells (Ha et al., 2019), and is a constituent of the SASP, which as previously described is a powerful driver of age-related dysfunction influencing important mediators of inflammaging (Coppé et al., 2010; Fujita et al., 2016). As various SASP circulating components have been shown to be strongly associated with advanced chronological age, the behavior of biomarkers such as GDF-15 thus support the hypothesis that the SASP could become an informative candidate biomarker of biological age, meaning its respective biological levels could be leveraged to predict risk for adverse health outcomes (Schafer et al., 2020).

Relevant to the age-related vascular dysfunction, it has been demonstrated that GDF-15 may play an important role in the initiation and progress of atherosclerosis exerting proinflammatory effects induced by the macrophage-produced monokines. Indeed, evidence has shown that GDF-15 has been identified in human carotid atherosclerotic plaques, and also co-localized in macrophages (Wiklund et al., 2010). In addition, early studies have indicated the association of GDF-15 with endothelial and myocardial dysfunction and atherosclerotic burden in the elderly after adjustment for conventional CV risk factors (Lind et al., 2009).

#### Association With Cardiovascular Disease

In addition, GDF-15 has shown a strong predictive ability of clinical outcomes across different CV risk populations. More specifically, GDF-15 has shown to predict all-cause and CV mortality in healthy populations free from CVD (Wiklund et al., 2010; Eggers et al., 2013), while in women, (but not men), with carotid atherosclerosis it has been shown to predict secondary CVD events (Gohar et al., 2017). Importantly, GDF-15 holds an established role as a predictor of future CV events and mortality in populations with CHD while it is a predictor of all-cause mortality and major CV events in patients with acute coronary syndromes (Hagström et al., 2016), and chronic heart failure (Kempf et al., 2007).

#### Measurement of Growth Differentiation Factor 15

Several biochemical approaches such as ELISA which allow for the measurement of plasma levels of GDF-15 in cost-effective ways which are easy to perform positions GDF-15 as a strong potential biomarker for the aging process and many age-related diseases (Barma et al., 2017; Conte M. et al., 2020) though considerations should be made with respect to the analyses obtained. According to a recently published study by Doerstling et al. (2018), a selected group of apparently healthy participants (*n* = 268), were analyzed for circulating GDF-15 using the generalized additive models for location scale and shape (GAMLSS) in order to develop age-dependent centile values. Unadjusted and adjusted COX proportional hazards models were used to assess the association between the derived GDF-15 reference values (expressed as centiles) and all-cause mortality. Smoothed centile curves showed increasing GDF-15 with age in the apparently healthy participants, while age-dependent GDF-15 centiles remained a significant predictor of all-cause mortality in all subsequent adjusted models.

### Epigenetic Based Circulating Biomarkers

Epigenetic regulation refers to modifications influencing gene expression independently of gene sequence. These changes mainly include DNA methylation, histone modifications, chromatin remodeling and non-coding RNA based gene regulation (López-Otín et al., 2013); all of which participate in the process of vascular aging and are closely relate to several CV risk factors including older age, high-salt and fat diet, smoking and sedentariness (Ding et al., 2018). Among all epigenetic mechanisms, DNA methylation is the most extensively studied.

#### Micro-Ribonucleic Acids

##### Pathophysiology

Micro-ribonucleic acids (miRNAs) are small, highly conserved non-coding RNA molecules involved in the regulation of gene expression (Table 2; Ambros, 2004). More than 2000 miRNAs have been discovered in humans that can regulate one third of the genes in the genome (Hammond, 2015).

**TABLE 2:** Significance of several microRNAs (*miRs*) in vascular aging process (based on a systematic review by Navickas et al., 2016).

Process	Type of microRNAs
Endothelial function and angiogenesis	*miR-1, miR-133*
Vascular smooth muscle cell differentiation	*miR-133, miR-145*
Communication between vascular smooth muscle and endothelial cell to stabilize plaques	*miR-145*
Apoptosis	*miR-1, miR-133, miR-499*
Cardiac myocyte differentiation	*miR-1, miR-133, miR-145, miR-208, miR-499*
Repression of cardiac hypertrophy	*miR-133*

##### Association With Physiological Aging and Vascular Markers

There is growing evidence that vascular aging is associated with a dysregulation in the expression of different microRNAs, which are involved in crucial pathogenetic mechanisms leading to impairment of angiogenic processes (Ungvari et al., 2013), decreased cellular stress resilience (Csiszar et al., 2014) and plaque formation, destabilization and rupture (Menghini et al., 2014; Ungvari et al., 2018). Hence, it has been observed that miRNA expression is decreased during cellular senescence, a hallmark of vascular aging, in human tissue culture cells grown *in vitro* (Brosh et al., 2008). Additionally, it has been shown that several miRNAs have similar expression patterns in both senescent cells and in aging human mononuclear cells and, most importantly, that there is an age-dependent decrease in miRNA expression in peripheral blood mononuclear cells (Hooten et al., 2013). However, existing evidence from larger population studies shows that the expression of particular miRNAs distinctly differs in relation to chronological age, with certain miRNAs showing a positive correlation with age while the majority of them being significantly downregulated in older compared to younger individuals (Meder et al., 2014; Ameling et al., 2015; Huan et al., 2018). Interestingly, while age clearly contributes to expression changes in miRNAs, gender seems to have a rather modest effect. In fact, most miRNAs show a similar behavior over the lifespan in males and females (Fehlmann et al., 2020).

There is increasing evidence confirming that miRNAs are highly influential in various physiological and pathological processes which mediate vascular function. For example, it has been demonstrated that miR-126 and miR-21 can mediate a significantly pro-inflammatory vascular phenotype including but not limited to endothelial cell specific inflammation, in addition to vascular smooth muscle cell proliferation and fibrosis (Lin et al., 2016). Similarly, certain miRNAs are recognized as active contributors to the pathogenesis of vascular calcification (Leopold, 2014) and the progression of atherosclerosis (Toyama et al., 2018). Finally, miRNAs such as miR-34a/b/c, miR-21, and miR-501-3p have been independently associated with established vascular markers of aging including PWV (Parthenakis et al., 2017; Toyama et al., 2018; Gatsiou et al., 2021) whereas miR-132 and miR-1 have been shown to predict carotid artery stiffness (Šatrauskienė et al., 2021).

##### Association With Cardiovascular Disease

So far, there is currently insufficient evidence to conclusively determine the incremental value and the significance of microRNAs as predictors of clinical outcomes related to vascular aging. However, there are a select number of studies in humans highlighting the significance of several distinct microRNA types in this context. For example, microRNA-208b appears to be an important predictor of mortality, even after adjustment for age and gender, microRNA-133a is related to all-cause mortality, and microRNA-133a, microRNA-499, and microRNA-208a/b are significant diagnostic and/or prognostic markers across different CVD progression stages (Navickas et al., 2016). Moreover, microRNA-199a-3p is a predictor of worsening renal function in acute heart failure patients (Bruno et al., 2016), the complex of five microRNAs (microRNA-106a-5p, -424-5p, let-7g-5p, -144-3p, and -660-5p) is associated with the risk of future myocardial infarction (Bye et al., 2016), and a set of seven microRNAs was recently identified to reliably predict CV death in patients with CHD (Karakas et al., 2017). It is possible that the levels, and respective activities of these particular microRNAs may be dysregulated at different stages of the progression of coronary artery disease (CAD).

According to Šatrauskienė et al. (2021), circulating levels of miR-1 and miR-133 correlated with arterial markers of subclinical atherosclerosis (cardio-ankle vascular index, augmentation index normalized to a heart rate of 75 bpm, aortic pulse wave velocity and carotid artery stiffness).

According to Kumar et al. (2020), circulating miR-21 (as well as miR-133b) were dysregulated in patients with CAD, but without MI. Both of these miRs showed association with CAD severity from subclinical atherosclerosis to acute coronary syndrome. Another study by Cengiz et al. (2015) showed that miR-21 was related to subclinical atherosclerosis in carotid arteries in hypertensive patients.

Moreover, Wang et al. (2019) demonstrated that higher plasma levels of circulating miR-208b and miR-499 were positively associated with the severity of CAD.

Different miRs may be dysregulated in patients without CV risk (at the same time it could be dysregulated in other conditions for example multiple sclerosis, oncological diseases). Considering that CVD are multifactorial diseases, analyses of the profile of miRNA panels may have a more substantial diagnostic or prognostic value than any single miRNA.

##### Measurements of Micro-Ribonucleic Acids

Plasma and serum miRNAs are the most suitable source for reproducible measurements in everyday clinical practice (Chen et al., 2009; Sato et al., 2009; Wang et al., 2011). MiRNAs are extremely stable and long-term storage or freeze-thaw cycles do not significantly affect them (de Lucia et al., 2017). Reverse transcription quantitative real-time polymerase chain reaction (PCR) is the most widely sensitive method for microRNA profiling (Pritchard et al., 2012), however, it is still unclear which is the best strategy for data normalization (Meyer et al., 2010; Kok et al., 2015; Marabita et al., 2016). Another issue is that cut-off values for miRNAs have not been established yet for almost all miRNAs, except miRNA-133a (Wang et al., 2013).

Human studies on circulating miRNAs represent an active research field, and examples exist in the literature showing their potential use as diagnostic biomarkers. Though several normalization strategies are proposed (Marabita et al., 2016), the provision of uniform procedural guidelines - more than a universal set of normalizers, may assist in obtaining reliable quantifications and comparisons of circulating miRNAs (Schwarzenbach et al., 2015).

#### DNA Methylation

##### Pathophysiology

DNA methylation is characterized by the transfer of a methyl group from *S*-adenyl methionine to the 5th carbon atom of cytosine by a DNA methyltransferase (DNMT), resulting in the formation of 5-methylcytosine (5mC). In human DNA, 5mC is found in approximately 1.5% of genomic DNA. The majority of DNA methylation occurs on cytosines that precede a guanine nucleotide or CpG regions that are short, interspersed GC-rich DNA sequences (Jeltsch and Gowher, 2019). Those CpG sites occur with high frequency in genomic regions called CpG islands which contain the majority of promoters located near the transcription start site of a gene. In normal cells, CpG islands are usually unmethylated regardless of their level of expression, however, the presence of multiple methylated CpG sites inside them causes stable silencing of genes, thus influencing gene expression (Schübeler, 2015).

##### Association With Physiological Aging and Vascular Markers

DNA methylation changes and age seem to be inextricably linked and a rich body of literature confirms that aging has a profound effect on genome-wide DNA methylation levels with almost ∼2% of the CpG sites showing changes in DNA methylation (Unnikrishnan et al., 2019). More specifically, it has been demonstrated that the global DNA methylation levels increase over the first few years of life and then decrease beginning in late adulthood. However, at specific loci a significant increase in variability of DNA methylation levels with age has been reported; either toward hypomethylation or hypermethylation, with a predisposition for the second (Jones et al., 2015). Most importantly, certain changes in the methylation of a few hundred CpG sites which are consistent across individuals, have been strongly associated with age to the extent that multiple prediction models have been invented to accurately predict the chronological age in humans, commonly termed as epigenetic clocks (Hannum et al., 2013; Horvath, 2013). These models can be nowadays applied across a broad spectrum of tissues and cells although the age prediction accuracy varies depending on the tissue type. Even so, epigenetic clocks represent certain functional age-related epigenetic changes that are common across individuals and are currently considered the best biomarkers for predicting mortality in humans. In addition, epigenetic clocks could help explain why some age-related phenotypes occur. In a prospective study of healthy individuals, it was shown that sites of age-associated-DNA methylation display a greater variability across women who developed cervical cancer within 3 years compared to those who remained healthy (Teschendorff et al., 2012). However, it is uncertain as to how well epigenetic clocks can predict biological age.

So far, no human data regarding the association of DNA methylation with markers of vascular stiffness exist.

##### Association With Cardiovascular Disease

Evidence of differential DNA methylation patterns has been already observed in various CVD states. For example, enhanced DNMT1 activity has been observed in patients with severe atherosclerotic disease and it correlates with increased levels of inflammatory cytokines (Yu et al., 2016). Similarly, a broad trend toward DNA hypermethylation associated with atherosclerotic plaque progression has been observed (Valencia-Morales et al., 2015; Gallego-Fabrega et al., 2020). In addition, increased DNA methylation levels have been documented in elderly patients with myocardial infarction and CHD and have shown a positive correlation with the degree of coronary atherosclerosis (Jiang et al., 2019).

Finally, DNA methylation changes are claimed to be an independent predictor of mortality similarly to other risk factors, such as hypertension and DM (Martella and Fisher, 2021). In a 10-year follow-up study of 832 participants at the age of 70 years, a strong correlation was observed between DNA methylation status and the incidence of CVD (Lind and Lind, 2018). In addition, in a large meta-analysis including 13,089 individuals, DNA methylation-based measures were found to predict all-cause mortality over and above chronological age and traditional risk factors (Chen et al., 2016).

##### Measurement of DNA Methylation

DNA methylation is predominantly measured in DNA extracted from peripheral blood cells/whole blood cells samples. In terms of technology, the Illumina 450K array is the current gold standard for DNA methylation measurement, but its genomic coverage may be limited especially concerning regions that may be crucial to aging such as repetitive elements and long non-coding RNAs.

### Telomere Length

#### Pathophysiology

Telomere length has steadily garnered interest as a potential marker of measuring and quantifying vascular aging in recent years. Telomeres are defined as non-coding, repetitive DNA sequences (hexanucleotide TTAGGG) which are found at each end of the chromosomes of eukaryotic cells (Blackburn, 1991), are highly conserved and, in total, amount to an estimated 11 to 15 kilobases (Moyzis et al., 1988; Blasco, 2005, 2007). Functionally, telomeres preserve the genomic integrity by acting as protective caps to the genetic material and preventing attrition (Blackburn, 2001; Blasco, 2005).

#### Association With Physiological Aging and Vascular Markers

Telomere shortening is a well-known hallmark of organismal aging, and an accelerated rate of telomere attrition is also a common feature of age-related diseases. That said, telomere length is currently recognized as a ‘biological clock’ of sorts, with a significant number of studies in the literature clearly documenting an inverse correlation with human chronological age (Willeit et al., 2010; Müezzinler et al., 2013).

In addition, short telomere length has been significantly associated with vascular markers of arterial stiffness and aging such as PWV, although these findings are not consistent across different sexes and aging groups (Benetos et al., 2001; Strazhesko et al., 2015). Although not in all (De Meyer et al., 2009), there are quite a few studies demonstrating a significant relationship between carotid atherosclerosis, as a robust marker of subclinical atherosclerosis, and short telomere length (Benetos et al., 2004; Panayiotou et al., 2010; Toupance et al., 2017).

#### Association With Cardiovascular Disease

Data have shown that short telomere length not only precedes the development of atherosclerotic CVD but may even play a pathogenetic role in the development of the disease later in life (Benetos et al., 2018; Huang et al., 2020). Recent meta-analyses have demonstrated that short telomere length is also significantly and independently associated with the risk of CHD (Haycock et al., 2014), and the presence of stroke and DM type 2 (D’Mello et al., 2014). Importantly, there is evidence that short telomere length is an independent predictor of future CV events namely myocardial infarction and stroke (Willeit et al., 2010). Furthermore, in a meta-analysis including 121,749 individuals, short telomere length was associated with increased all-cause mortality risk in the general population (Wang et al., 2018).

#### Measurement of Telomeres

Telomere length is predominantly measured in DNA extracted from peripheral blood leukocytes. Currently, there are three main methods for measuring telomere length in said DNA: (a) Southern blot (Lin and Yan, 2005), (b) quantitative polymerase chain reaction (Lin and Yan, 2005), and (c) fluorescent *in situ* hybridization-based methods (Lansdorp et al., 1996; Saldanha et al., 2003). Each method presents its own unique advantages and disadvantages, depending on the type of analyses required, and the scale of study being devised. Overall, the Southern blot remains the gold standard for measuring telomere length. Despite being the first and oldest of the three methods, it remains the most accurate, and thus, it is utilized by those interested in precise measurement of telomere length. However, it is the most time-consuming method of the three, and also does not allow for the analysis of individual chromosomes or even cells (Saldanha et al., 2003; Aviv et al., 2011). qPCR on the other hand, while less accurate, does allow for single cell/chromosome analyses owing to its sensitivity and dynamic range enabling it to work with minute amounts of material. As such, qPCR is often the most employed method for large population-based studies owing to the nature of analyses available, and the efficiency in doing such (Cawthon, 2002; Saldanha et al., 2003).

## Conclusion

The term biological marker or biomarker refers to any substance, structure or process that can be measured in the human body, and influences or predicts the incidence of outcomes or diseases (The World Health Organization definition). Many candidate biomarkers have been put forward as viable indicators or vascular aging based on current understanding of vascular pathophysiology, and have been scrutinized accordingly, however, none to date have satisfied all necessary criteria to be translated effectively into clinical practice. While genetic and imaging biomarkers represent some of the current practices, identifying an appropriate circulating biomarker/s, that is/are easily measured in blood or in urine, could be an incredibly useful and direct means of determining different phases of the vascular aging and in turn CV disease susceptibility/progression. While technology and approaches allow for the quick and easy measurement of circulating biomarkers, the acquisition and handling of the body fluids containing them present many limitations; such as sensitivity to temperature, rapid blood coagulation and unstability of urine proteins that are the result of renal filtration.

Our choice of circulating biomarkers which are understood and recognized to see their levels change with respect to indices of vascular aging, was derived from the long list of most frequently mentioned in current literature data (Supplementary Tables S1, S2), and includes those associated with oxidative stress, inflammation, the extracellular cell matrix, and epigenetics, amongst others. Based on the data presented in this review, it is unlikely that a single molecule will ever be considered adequate for most conditions. In that regard, the new paradigm is the development of diagnostic panels of biomarkers. In this process, we must progress our understanding of existing biomarkers, in addition to identifying new biomarkers, and evaluate their real clinical relevance in addition to embracing new technologies in the fields of proteomics, genomics, metabolomics, lipidomics and bioinformatics to achieve this.

## Author Contributions

All authors reviewed the relevant literature and drafted the initial manuscript. KG, DT-P, AŠ, JZ, MB-L, and JN made a choice of biomarkers. EG and AL prepared the figure. EF, KR, ÖA, AL, and PN contributed additional material to the manuscript. DT-P, KG, EG, and JN supervised the study and oversaw preparation, editing, and revision of the manuscript. RC, RB, and KR reviewed the manuscript. All authors have read and approved the final manuscript.

## Conflict of Interest

The authors declare that the research was conducted in the absence of any commercial or financial relationships that could be construed as a potential conflict of interest.

## Publisher’s Note

All claims expressed in this article are solely those of the authors and do not necessarily represent those of their affiliated organizations, or those of the publisher, the editors and the reviewers. Any product that may be evaluated in this article, or claim that may be made by its manufacturer, is not guaranteed or endorsed by the publisher.

## Footnotes

1
www.vascagenet.eu
