# Supplementary material for: Pathophysiology of Circulating Biomarkers and Relationship With Vascular Aging: A Review of the Literature From VascAgeNet Group on Circulating Biomarkers, European Cooperation in Science and Technology Action 18216
Source: Front Physiol. 2021 Dec 14;12:789690. doi: 10.3389/fphys.2021.789690 (PMC8712891; doi:10.3389/fphys.2021.789690)
Supplement: Supplementary file 1 [file Table_1.pdf]

Supplementary Material

Table S1. Extensive list of circulating biomarkers related to vascular aging, most frequently mentioned in available literature.

**Biomarker**	**hs-CRP**	**IL-1 beta, IL-2, IL-6**	**TNF-alpha receptor 2**	**MMP 1, 2, 3 9, 12**	**Cystatin-C**	**GDF-15**	**IGF-1**	**Epigenetic markers**<br>miR-145,<br>miR-29,<br>miR-765 ect.
Underlying mechanism related to vascular aging	Inflammation	Inflammation	Inflammation	Matrix	Kidney aging	Stress response and mitochondria	Nutrient signaling	Epigenetic
Diseases	CVD, cancer,<br>inflammatory diseases	CVD: MI, obesity, diabetes; cancer, MCI/dementia, stoke, inflammatory diseases	CVD, cancer, MCI/dementia	CVD: hypertension, arterial aneurysm/ dissection, AF; cancer	CKD, CVD	CVD: heart failure, MI, atrial fibrillation, stroke; cancer, dementia, metabolic syndrome, diabetes, inflammation,	CVD, cancer, brain diseases	Cancer, CVD

						CKD		
Proof of concept	+++	+++	+++	+++	+++	+++	++	+++
Prospective validation	+++<br>(CRP - independently associated with mortality risk) 1	+++<br>(IL-6 - independently associated with mortality risk) 2<br><br>(IL-6 predicts post-acute MI heart failure) 3	+++<br>(TNF - alpha - independently associated with mortality risk) 2	++<br>(MMP-9 level is an independent predictor of recurrent arrhythmia after catheter ablation in patients with persistent AF) 4<br><br>(MMP-3 baseline level in patients with history of CAD is a potential predictor for cardiovascular outcomes) 5	+++<br>(strong and independent risk factor for all-cause and cause-specific mortality in the elderly) 6,7	+++<br>(association with risk of mortality, cardiovascular disease, and heart failure, and biology of aging) 2	+<br>(relation between IGF-1 and mortality/frailty is U shaped with both high and low levels associated with all-cause mortality and adverse health outcomes) 2	++/+++<br>(miR-208b: significant mortality predicting biomarker, which remained significant after adjustment for age and gender<br><br>miR-133a: significantly related to all-cause mortality<br><br>miR-133a, miR-499, miR-208a/b: significant diagnostic and/or prognostic markers across different cardiovascular disease progression stage) 10
Incremental value	++	++	++	++	++	++	+	++
Clinical utility	+++	/	/	/	/	/	/	/
Clinical outcomes	++	/	/	++	/	/	/	+

Cost-effectiveness	+	+	/	+	+	+	/	+
Ease of use	++++	++++	+++	/	+++	++	/	+++ (Serum and plasma miRNAs are probably the most suitable source for clinical application as sample collection is quick, simple, and reproducible)
Methodological consensus	++++	++ (Il-2, Il-1β demonstrate inconsistent detectability resulting from analyte degradation in long-term storage, or low assay sensitivity 11 (ELISA)	++ (serum TNFα serum levels tend to be low and unstable with storage at − 80 °C) (ELISA)	++ (zymography and immunoassays)	++++ (measured by BNII nephelometer that utilizes a particle-enhanced immunonepholometric assay (N Latex Cystatin-C)	+++ (measured with a research ELISA, a research IRMA, an ELISA using antibodies from R&D Systems (now marketed as a Quantikine® ELISA), and a Luminex sandwich assay developed by Alere; concentrations correlate well) 8	++ (ELISA, radioimmunoassay, immunoradiometric assay)	+++ (miRNAs are simply quantified by real-time polymerase chain reaction or microarrays) (miRNAs are remarkably stable and resistant in the blood and long-term storage or freezing/thawing cycles) (it is still unclear what is the best strategy for data normalization)
Reference values (or cut-off values)	Cut-off value: 2 mg/L 1	/	/	Cut-off value: MMP-3 > 9.3 ng/mL (patients with CAD had lower survival rate) 5	/	Cut-off value: 1560 ng/L (for all-cause mortality in patients with suspected MI) 9	/	(Only MiR-133a) 12

Abbreviations: hs-CRP - high sensitivity C-reactive protein; Il - interleukin; TNF alpha - tumor necrosis factor alpha; MMP - matrix metalloproteinases; GDF-15 - growth differentiation factor 15; IGF-1 - insulin-like growth factor 1; CKD - chronic kidney disease; CVD - cardiovascular disease; MI - myocardial infarction; MCI - mild cognitive impairment; CAD-cardiovascular disease, AF - atrial fibrillation; ++++ - strongly supported by literature; +++ - supported by literature; ++ - no consistency in literature; + - insufficient data; / - no data.

References

1. Vlachopoulos C, Xaplanteris P, Aboyans V, Brodmann M, Cífková R, Cosentino F, et al. The role of vascular biomarkers for primary and secondary prevention. A position paper from the European Society of Cardiology Working Group on peripheral circulation: Endorsed by the Association for Research into Arterial Structure and Physiology (ARTERY) Society. Atherosclerosis (2015) Aug;241(2):507-32. doi: 10.1016/j.atherosclerosis.2015.05.007.
2. Justice JN, Ferrucci L, Newman AB, Aroda VR, Bahnson JL, Divers J, et al. A framework for selection of blood-based biomarkers for geroscience-guided clinical trials: report from the TAME Biomarkers Workgroup. Geroscience (2018) Dec;40(5-6):419-436. https://doi.org/10.1007/s11357-018-0042-y
3. Lino DOC, Freitas IA, Meneses GC, Martins AMC, Daher EF, Rocha JHC, et al. Interleukin-6 and adhesion molecules VCAM-1 and ICAM-1 as biomarkers of post-acute myocardial infarction heart failure. Braz J Med Biol Res (2019) Nov 25;52(12):e8658. doi: 10.1590/1414-431X20198658.
4. Wu G, Wang S, Cheng M, Peng B, Liang J, Huang H, Jiang X, et al. The serum matrix metalloproteinase-9 level is an independent predictor of recurrence after ablation of persistent atrial fibrillation. Clinics (2016) 71(5):251-6. https://doi.org/10.6061/clinics/2016(05)02
5. Guizani I, Zidi W, Zayani Y, Boudiche S, Hadj-Taieb S, Sanhaji H, et al. Matrix metalloproteinase-3 predicts clinical cardiovascular outcomes in patients with coronary artery disease: a 5 years cohort study. Mol Biol Rep (2019) Oct;46(5):4699-4707. doi: 10.1007/s11033-019-04914-4.
6. Shlipak MG, Wassel Fyr CL, Chertow GM, Harris TB, Kritchevsky SB, Tylavsky FA, et al. Cystatin C and mortality risk in the elderly: the health, aging, and body composition study. J Am Soc Nephrol (2006) Jan;17(1):254-61. doi: 10.1681/ASN.2005050545.
7. Bevc S, Hojs N, Knehtl M, Ekart R, Hojs R. Cystatin C as a predictor of mortality in elderly patients with chronic kidney disease. Aging Male (2019) Mar;22(1):62-67. doi: 10.1080/13685538.2018.1479386.
8. Wollert KC, Kempf T, Wallentin L. Growth Differentiation Factor 15 as a Biomarker in Cardiovascular Disease. Clin Chem (2017) Jan;63(1):140-151. doi: 10.1373/clinchem.2016.255174.
9. Walter J, Nestelberger T, Boeddinghaus J, Twerenbold R, Croton L, Badertscher P, et al. Growth differentiation factor-15 and all-cause mortality in patients with suspected myocardial infarction. Int J Cardiol (2019) Oct 1;292:241-245. doi: 10.1016/j.ijcard.2019.04.088.
10. Navickas R, Gal D, Laucevičius A, Taparauskaitė A, Zdanytė M, Holvoet P. Identifying circulating microRNAs as biomarkers of cardiovascular disease: a systematic review. Cardiovasc Res (2016) Sep;111(4):322-37. doi: 10.1093/cvr/cvw174.
11. McKay HS, Margolick JB, Martínez-Maza O, Lopez J, Phair J, Rappocciolo G, Denny TN, Magpantay LI, Jacobson LP, Bream JH. Multiplex assay reliability and long-term intra-individual variation of serologic inflammatory biomarkers. Cytokine. (2017) Feb;90:185-192. doi: 10.1016/j.cyto.2016.09.018. Wang
12. Long G, Zhao C, Li H, Chaugai S, Wang Y, et al. Plasma microRNA-133a is a new marker for both acute myocardial infarction and underlying coronary artery stenosis. J Transl Med (2013) Sep 23;11:222. doi: 10.1186/1479-5876-11-222.
