# Supplementary material for: Pathophysiology of Circulating Biomarkers and Relationship With Vascular Aging: A Review of the Literature From VascAgeNet Group on Circulating Biomarkers, European Cooperation in Science and Technology Action 18216
Source: Front Physiol. 2021 Dec 14;12:789690. doi: 10.3389/fphys.2021.789690 (PMC8712891; doi:10.3389/fphys.2021.789690)
Supplement: Supplementary file 2 [file Table_2.pdf]

Supplementary Material

Table S2. Extensive list of circulating biomarkers related to vascular aging, less frequently mentioned in available literature

**Biomarker**	**Telomere length, dysfunction, uncapping**	**ANG II**	**MCP-1**	**sVCAM-1**	**SIRT 1 (and SIRT 6)**	**mTOR**	**AMPK**
Underlying mechanism related to vascular aging	Genetics	Matrix	Inflammation	Angiogenesis, cell adhesion, inflammation	Mitochondrial biogenesis, metabolism<br>protective role against inflammation, vascular aging, heart disease, and atherosclerotic plaque development	Metabolism<br>promote angiogenesis and limit acute cell death to foster cardiac repair and tissue regeneration<br>oxidative stress, aging, proliferative disorders, and metabolic abnormalities	Metabolism
Diseases	Obesity, diabetes, coronary heart disease, arterial hypertension, aortic aneurysm, MI, stroke	CVD: arterial hypertension, CAD, MI, stroke, left ventricular hypertrophy, arrhythmias; type 2 diabetes 4,5	CVD: ACS (formation, progression, and destabilization of atheromatous plaques, post-infarction remodeling), aortic stenosis; psoriasis, rheumatoid arthritis, atherosclerosis, neuroinflammation,	CAD, type 2 diabetes 8; post-acute MI heart failure 9, atrial fibrillation 10	Cancer, CVD: atherosclerosis, MI, cardiac hypertrophy, heart failure; metabolic diseases: metabolic syndrome, type 2 diabetes, age-related neurodegenerative diseases, stress resistance, vascular aging (involved in the cellular events controlling aging-related disorder) 12	CVD, cardiac hypertrophy, cancer, obesity, diabetes, neurodegenerative disorders 15; autoimmune diseases 16	CVD: heart failure, MI, cardiomyopathies 17; metabolic disorders: diabetes, obesity 18; stroke, cognitive impairment 19; cancer 20

			ulcerative colitis				
Proof of concept	+++	+++	+++	+++	+++	+	+ (evidence in animal models, data with humans are inconsistent)
Prospective validation	++ (short telomere length is associated with an increased risk of MI and hypertension 1,2 (telomere length is inconsistently or not associated with clinical or functional outcomes) 3	++ (among patients with hypertension, raised levels of ANG II were predictive of MI) 4	++ (MCP-1 plasma levels have prognostic value in the acute and chronic phase following ACS) 6 (associated with subclinical atherosclerotic disease severity in postmenopausal women without CVD) 7	++ (VCAM-1 predicts post-acute MI heart failure) 9 (sVCAM-1 concentration in the pre-operative serum of patients undergoing CABG may accurately predict the onset of de novo postoperative AF) 10	++ (SIRT1 activity is positively correlated with serum triglyceride in men, and with waist circumference, systolic blood pressure, diastolic blood pressure, and serum triglyceride in women) 13 (serum SIRT1 may be a potentially new biomarker for T2DM) 14	+	+ (few clinical studies with AMPK activators)
Incremental value	++	++	++	+++	++	+	/
Clinical utility	/	+	+	++ (VCAM-1 can sustain high-risk carotid plaques and may be useful for identifying patients in whom more aggressive treatment is warranted) 11	+	+	+

Clinical outcomes	/	/	/	/	+	+	/
Cost-effectiveness	/	/	/	/	/	/	/
Ease of use	+	/	+	+	+	+	+
Methodological consensus	++ Southern blot (which is expensive and time consuming), real-time PCR (which is more suitable for epidemiological studies due to the ability to standardize results), and fluorescence in-situ hybridization. Methods are not well standardized and comparable.	++ (ELISA)	++ (enzyme-linked immunosorbent assay - ELISA)	++ (ELISA)	++ (enzyme-linked immunosorbent assay (ELISA), Western blot)	/	++ (immunoblotting)
Reference values (or cut-off values)	/	/	/	/	/	/	/

Abbreviations: ACS - acute coronary syndrome; AF - atrial fibrillation; AMPK - 5′ AMP-activated protein kinase; ANG II - angiotensin receptor II; CABG - coronary artery bypass grafting; CAD - coronary artery disease; CVD - cardiovascular disease; MCP-1 - monocyte chemoattractant protein-1; MI - myocardial infarction; mTOR - mechanistic/mammalian target of rapamycin; SIRT 1 - sirtuin 1; sVCAM-1

- soluble vascular cell adhesion molecule 1; ++++ - strongly supported by literature; +++ - supported by literature; ++ - no consistency in literature; + - insufficient data; / - no data.

References

1. Zee RY, Michaud SE, Germer S, Ridker PM. Association of shorter mean telomere length with risk of incident myocardial infarction: a prospective, nested case-control approach. Clin Chim Acta (2009) May;403(1-2):139-41. doi: 10.1016/j.cca.2009.02.004.
2. Nilsson PM, Dahlman D, Roos G, Nordfjäll K, Melander O. Telomere attrition over fifteen years is associated with clinical markers of cardiovascular ageing in middle-aged subjects – The Malmo Diet and Cancer Study. J Hypertens (2011) 29. e475. 10.1097/00004872-201106001-01434.
3. Justice JN, Ferrucci L, Newman AB, Aroda VR, Bahnson JL, Divers J, et al. A framework for selection of blood-based biomarkers for geroscience-guided clinical trials: report from the TAME Biomarkers Workgroup. Geroscience (2018) Dec;40(5-6):419-436. doi: 10.1007/s11357-018-0042-y.
4. Patel JV, Lim HS, Varughese GI, Hughes EA, Lip GY. Angiopoietin-2 levels as a biomarker of cardiovascular risk in patients with hypertension. Ann Med (2008) 40(3):215-22. doi: 10.1080/07853890701779586.
5. Gavras I, Gavras H. Angiotensin II as a cardiovascular risk factor. J Hum Hypertens (2002) May;16 Suppl 2:S2-6. doi: 10.1038/sj.jhh.1001392.
6. Gonzalez-Quesada C, Frangogiannis NG. Monocyte chemoattractant protein-1/CCL2 as a biomarker in acute coronary syndromes. Curr Atheroscler Rep (2009) Mar;11(2):131-8. doi: 10.1007/s11883-009-0021-y.
7. Basurto L, Gregory MA, Hernández SB, Sánchez-Huerta L, Martínez AD, Manuel-Apolinar L, et al. Monocyte chemoattractant protein-1 (MCP-1) and fibroblast growth factor-21 (FGF-21) as biomarkers of subclinical atherosclerosis in women. Exp Gerontol (2019) Sep;124:110624. doi: 10.1016/j.exger.2019.05.013.
8. Moradi N, Fadaei R, Emamgholipour S, Kazemian E, Panahi G, Vahedi S, et al. Association of circulating CTRP9 with soluble adhesion molecules and inflammatory markers in patients with type 2 diabetes mellitus and coronary artery disease. PLoS One (2018) Jan 30;13(1):e0192159. doi: 10.1371/journal.pone.0192159.
9. Lino DOC, Freitas IA, Meneses GC, Martins AMC, Daher EF, Rocha JHC, et al. Interleukin-6 and adhesion molecules VCAM-1 and ICAM-1 as biomarkers of post-acute myocardial infarction heart failure. Braz J Med Biol Res (2019) Nov 25;52(12):e8658. doi: 10.1590/1414-431X20198658.
10. Harling L, Lambert J, Ashrafian H, Darzi A, Gooderham NJ, Athanasiou T. Pre-operative serum VCAM-1 as a biomarker of atrial fibrillation after coronary artery bypass grafting. J Cardiothorac Surg (2017) Aug 18;12(1):70. doi: 10.1186/s13019-017-0632-2.
11. Weinkauf CC, Concha-Moore K, Lindner JR, Marinelli ER, Hadinger KP, Bhattacharjee S, et al. Endothelial vascular cell adhesion molecule 1 is a marker for high-risk carotid plaques and target for ultrasound molecular imaging. J Vasc Surg (2018) Dec;68(6S):105S-113S. doi:10.1016/j.jvs.2017.10.088.
12. D'Onofrio N, Servillo L, Balestrieri ML. SIRT1 and SIRT6 Signaling Pathways in Cardiovascular Disease Protection. Antioxid Redox Signal (2018) Mar 10;28(8):711-732. doi: 10.1089/ars.2017.7178.
13. Lee HJ, Yang SJ. Aging-Related Correlation between Serum Sirtuin 1 Activities and Basal Metabolic Rate in Women, but not in Men. Clin Nutr Res (2017) Jan;6(1):18-26. doi: 10.7762/cnr.2017.6.1.18.
14. Gok O, Karaali Z, Ergen A, Ekmekci SS, Abaci N. Serum sirtuin 1 protein as a potential biomarker for type 2 diabetes: Increased expression of sirtuin 1 and the correlation with microRNAs. J Res Med Sci (2019) Jun 25;24:56. doi: 10.4103/jrms.JRMS_921_18.
15. Das A, Reis F, Mishra PK. mTOR Signaling in Cardiometabolic Disease, Cancer, and Aging 2018. Oxid Med Cell Longev (2019) Feb 4;2019:9692528. doi: 10.1155/2019/9692528.
16. Perl A. mTOR activation is a biomarker and a central pathway to autoimmune disorders, cancer, obesity, and aging. Ann N Y Acad Sci (2015) Jun;1346(1):33-44. doi: 10.1111/nyas.12756.

17. Li T, Mu N, Yin Y, Yu L, Ma H. Targeting AMP-Activated Protein Kinase in Aging-Related Cardiovascular Diseases. Aging Dis (2020) 11(4):967-977. doi:10.14336/AD.2019.0901
18. Ruderman NB, Carling D, Prentki M, Cacicedo JM. AMPK, insulin resistance, and the metabolic syndrome. J Clin Invest (2013) Jul;123(7):2764-72. doi: 10.1172/JCI67227.
19. Kim E, Lee SH, Lee KS, Cheong HK, Namkoong K, Hong CH, et al. AMPK γ2 subunit gene PRKAG2 polymorphism associated with cognitive impairment as well as diabetes in old age. Psychoneuroendocrinology (2012) Mar;37(3):358-65. doi: 10.1016/j.psyneuen.2011.07.005.
20. Cheng J, Zhang T, Ji H, Tao K, Guo J, Wei W. Functional characterization of AMP-activated protein kinase signaling in tumorigenesis. Biochim Biophys Acta (2016) Dec;1866(2):232-251. doi: 10.1016/j.bbcan.2016.09.006.

frontiers
